# The clinical value of acupuncture combined with Wenjing decoction in treating primary dysmenorrhea in women—a systematic review and meta-analysis of randomized controlled trials

**DOI:** 10.3389/fmed.2025.1650158

**Published:** 2025-09-08

**Authors:** Mei Zhao, Fuhua Zhou, Chunmei Qiu, Xiumei Hu, Caiping Xie, Lixin Wen, Lianwei Xu

**Affiliations:** ^1^Sanming Hospital of Integrated Medicine, Sanming, China; ^2^Department of Gynecology, Longhua Hospital, Shanghai University of Traditional Chinese Medicine, Shanghai, China

**Keywords:** primary dysmenorrhea, Wenjing decoction, acupuncture therapy, meta-analysis, integrative medicine

## Abstract

**Objective:**

To systematically evaluate the clinical efficacy of combining acupuncture and Wenjing decoction for primary dysmenorrhea (PD) of cold congealing and blood stasis type, using the meta-analysis research methods, to provide references for clinical treatment.

**Methods:**

A randomized controlled trial (RCT) on the treatment of PD using Wenjing decoction with or without acupuncture was searched across multiple databases from 1 January 2000 to 1 March 2025. Outcome measurements included serum hormone levels, pain scores, uterine artery blood flow, and overall effectiveness rate. The quality of the studies included was assessed using the risk of bias tool. The methodological quality of the included studies was assessed with the Cochrane Collaboration tool. Review Manager 5.4 software was employed for quantitative synthesis, and funnel plots were utilized to evaluate potential reporting bias.

**Results:**

A total of 11 RCTs with 1,024 patients with primary dysmenorrhea were included. Wenjing decoction combined with acupuncture in the treatment of PD has obvious advantages over the control group in improving the effective rate [relative risk (RR) = 1.24], [95% confidence interval (CI): (1.06, 1.42), I^2^ = 34%, *p* = 0.15], increasing the level of PGE₂ [mean difference (MD) = 5.9, 95% CI: (2.27, 9.52), I^2^ = 63%, *p* = 0.07], reducing the level of PGF_2α_ [MD = −11.96, 95% CI: (−25.21, −1.28), I^2^ = 63%, *p* = 0.07], reducing pain score [−4.13, 95% CI: (−6.42, −1.84), I^2^ = 99%, *p* < 0.00001], improving uterine artery blood flow resistance index (RI) [MD = −0.09, 95% CI: (−0.12, −0.07), I^2^ = 18%, *p* = 0.27], and uterine artery blood flow pulsatility index (PI) [MD = −0.39, 95% CI: (−0.44, −0.33), I^2^ = 0%, *p* = 0.41]. The differences are statistically significant (*p* < 0.05).

**Conclusion:**

The combination of acupuncture and Wenjing decoction in the treatment of primary dysmenorrhea (PD) is superior to simple Western medicine treatment in reducing the level of prostaglandin F2 alpha (PGF_2α_), increasing the level of prostaglandin E2 (PGE2), decreasing the pain score, improving the resistance index (RI) and pulsatility index (PI) of uterine artery blood flow, as well as the total effective rate. However, the methodological quality of the studies included was undesirable, necessitating further verification with more well-designed and high-quality multicenter RCTs.

**Systematic review registration:**

https://www.crd.york.ac.uk/prospero/, identifier CRD42024623207.

## Introduction

Primary dysmenorrhea (PD) is more common in adolescent women, with menstrual tingling, dull pain, and cramping in the lower abdomen are the main symptoms, accompanied by fatigue, nausea, vomiting, backache, and other related symptoms, which cause significant discomfort to the patient (1, 2). The endometrium synthesizes PG, which converts anthoritic tetraenoic acid (AA) into inflammatory metabolites such as PGF_2α_ and PGE_2_ in response to cyclooxygenase-2 (COX-2). Among them, the increase in PGF_2α_ content can cause excessive uterine contractions, restrict blood flow, and induce the production of anaerobic metabolites, which stimulate pain receptors and thereby trigger PD (3). Estradiol (E2) and progesterone (P) are both hormones synthesized by the ovaries, and modern studies have shown that (4) the occurrence of dysmenorrhea is significantly related to sex hormones, and when E_2_ is elevated, it can indirectly promote the production and release of PGF_2α_, thereby aggravating dysmenorrhea. Oxytocin (OT) can affect dysmenorrhea, and studies have found that the level of OT in patients with dysmenorrhea is much higher than that in non-menstruating women, and the degree of abdominal pain in patients with primary dysmenorrhea is directly proportional to the amount of OT (5). Vasopressin (arginine vasopressin (AVP)) is secreted during uterine smooth muscle contraction and can be combined with prostaglandin (PG) to promote myometrium sensitivity to oxytocin, thereby aggravating dysmenorrhea (6). In recent years, many studies have found that PD is related to immune factors, and patients with primary dysmenorrhea are generally immunocompromised. Some scholars (7) have found that the cluster of differentiation 4 (CD4)/CD8 ratio of patients with primary dysmenorrhea is lower than that of healthy women, with a higher CD8 count. It is also related to abnormal blood flow in the uterine artery (8), inflammatory factors (9), and psychological factors (10), etc.

To the best of our knowledge, this is the first systematic review and meta-analysis specifically designed to evaluate the combined efficacy of acupuncture and Wenjing decoction for primary dysmenorrhea. While previous meta-analyses have examined acupuncture or Chinese herbal medicine separately, none have quantitatively synthesized evidence for this specific combination therapy. Our study aims to fill this evidence gap by providing a comprehensive assessment of its clinical value, mechanistic outcomes, and safety profile, thereby offering novel insights into integrative treatment approaches for PD.

Non-steroidal anti-inflammatory drugs (NSAIDs) are the first-line drugs for the treatment of PD (11). Although NSAIDs have a good analgesic effect on most patients with PD, there are still patients who are ineffective, and gastrointestinal reactions such as nausea and vomiting are apparent, which limits their clinical application to a certain extent (12). In addition, the most commonly used hormonal drugs for the treatment of PD are short-acting contraceptives. The primary physiological effects of OCPs include inhibition of the hypothalamic–pituitary–ovarian axis, inhibition of ovulation, and prevention of endometrial hyperplasia, thereby reducing the release of PGF_2α_ from the endometrium during menstruation and relieving spasmodic contraction of the uterus (13). For women who do not respond well to NSAIDs or who require contraception, ovulation suppression is an effective treatment for PD. However, oral contraceptives are poorly tolerated by many patients, regardless of the side effects of weight gain, pregnancy-like reaction, and decreased menstrual bleeding.

PD belongs to the category of “meridian abdominal pain” in traditional Chinese Medicine (TCM), which is related to cold pathogen injuring yang and Qi-blood obstruction, with cold coagulation and blood stasis as the main syndrome type, and warm meridian to dissipate cold, blood circulation, and blood stasis as the main treatment. Recent studies have demonstrated that TCM offers certain advantages in the treatment of primary dysmenorrhea through personalized syndrome differentiation diagnosis and treatment plans, as well as the treatment characteristics of multiple targets and multiple pathways (14, 15). As an important part of TCM comprehensive treatment, acupuncture has been widely recognized in the treatment of gynecological diseases. Acupuncture and moxibustion have the functions of reconciling yin and yang, strengthening the right, dispelling evil, and dredging the meridians. In this study, a meta-analysis was conducted to evaluate the efficacy of Wenjingtang combined with acupuncture in the treatment of PD, providing evidence-based support for the diversity of clinical treatment modalities and increasing clinical efficacy.

## Materials and methods

This meta-analysis was conducted in accordance with the guidelines of the Cochrane Handbook for Systematic Reviews of Interventions and the Preferred Reporting Items for Systematic Reviews and Meta-Analyses (PRISMA) (16), using the RevMan software (version 5.4; the Cochrane Collaboration, NCC, Copenhagen, Denmark).

Additionally, the protocol was registered and published on PROSPERO (PROSPERO CRD 42024623207).

### Search strategy and study selection

Seven databases were comprehensively searched, namely, the English-language databases Cochrane Library, Web of Science, EMBASE, PubMed, and the Chinese-language databases Wanfang, VIP Information, CBM, and China National Knowledge Infrastructure (CNKI) from inception up to December 2024. Randomized controlled trials on the treatment of PD by warm decoction combined with acupuncture are retrieved from these databases. The retrieval method combines subject terms and free terms. Our retrieval strategy comprised three main components: clinical conditions (including pain, menstrual, menstrual pain, menstrual pains, pains, menstrual, dysmenorrhea, menstrual pain, painful menstruation, period pain, painful period, menstrual cramps, menstrual disorder, pelvic pain, and painful menstrual periods); interventions (Wenjing combined with acupuncture, electroacupuncture, manual acupuncture, warming needle, acupuncture therapy, needling, needles, needle therapy, and moxibustion treatment); and study types (RCT).

No retrieval filters or limits were applied. To identify redundant papers, researchers manually examined the reference summaries of the retrieved articles. The initial screening of articles, conducted independently by the first two authors (MZ and LXW), involved a thorough review of titles, abstracts, and full texts to substantiate the eligibility of the studies. Any uncertainties regarding inclusion were deliberated among the other authors (LWX and FHZ).

### Inclusion and exclusion criteria

The inclusion criteria, based on the PICOS (patients, intervention, comparator, outcomes, and study design) framework, were pre-specified as: (1) Participants: patients diagnosed with dysmenorrhea of any age, case source, and disease duration and severity; (2) Intervention: Treatment with Wenjing decoction combined with Acupuncture; (3) Control method: Treatment with Western medicine; (4) Outcome measures with sufficient data: Effective rate; dysmenorrhea symptom score; serum PGF_2α_ and PGE2; uterine artery blood flow RI and PI, etc. (5) study type: RCT; and (6) availability of complete data in the literature and precise data in the experimental and control groups.

Exclusion criteria filtered out studies were (1) non-RCT, animal studies, case reports, conference proceedings, or literature reviews; (2) with ambiguous diagnostics; (3) of incomplete data or unavailable full-text; or (4) of duplicates.

### Data extraction and quality evaluation

Two independent reviewers (MZ and LXW) extracted the following data, such as the first author’s name, year of publication, study design, participants’ characteristics, specifics of Wenjing decoction combined with acupuncture and control intervention, and outcomes metrics. Disputes were resolved by a third reviewer (LXW).

Meanwhile, the quality assessment of the included studies was carried out by two independent reviewers (MZ and LXW) utilizing the Cochrane Collaboration’s Risk of Bias tool, and seven reviews were shared: (1) random sequence generation; (2) assign hidden schemes; (3) whether participants and intervention implementers were blinded; (4) blinding and attrition bias for outcome evaluation; and (5) whether the outcome data are complete; (6) selective reporting of research results; and (7) other sources of bias. Finally, three grades (low risk, unknown risk, and high risk) were evaluated according to the literature report. Any discrepancies were resolved through discussion with LXW.

### GRADE evidence grading evaluation

We evaluated the quality of evidence for all primary outcomes using the Grading of Recommendations Assessment, Development and Evaluation (GRADE) approach. This systematic methodology assesses evidence certainty across five domains: (1) risk of bias (study limitations), (2) inconsistency (heterogeneity), (3) indirectness (generalizability), (4) imprecision (sample size and confidence intervals), and (5) publication bias. Randomized controlled trials initially start as high-quality evidence but may be downgraded based on deficiencies in these domains. Conversely, evidence may be strengthened for large effect sizes, dose–response gradients, or when residual confounding would likely reduce the observed effect. Two independent reviewers (MZ and LXW) conducted the GRADE assessments, with any discrepancies resolved through discussion with a third reviewer (LWX). The final evidence certainty was categorized into four levels: high (further research is improbable to change our confidence in the effect estimate), moderate (further research may have an important impact), low (further research is very likely to have an important impact), or very low (any effect estimate is very uncertain).

### Statistical analysis

Use RevMan 5.4 software to conduct heterogeneity tests, draw forest plots, and funnel plots. For dichotomous variables, the relative risk (RR) is used to represent the effect index, and for continuous variables, the mean difference (MD) or standardized mean difference (SMD) is used to measure and represent the effect index. The chi-square test and I^2^ statistic are used to judge the heterogeneity among studies. If the heterogeneity among studies has no statistical significance (*p* > 0.1 and I^2^ < 50%), the fixed effects model is adopted; otherwise, the random effects model is used.

Sensitivity analysis is carried out by removing individual literatures one by one, and subgroup analysis is also used for heterogeneity testing. Publication bias is evaluated by drawing funnel plots.

Planned Subgroup Analyses and Meta-Regression: To investigate potential sources of heterogeneity, we pre-specified the following subgroup analyses if sufficient data were available (≥10 studies per subgroup):

1. Type of Acupuncture: manual acupuncture vs. electroacupuncture vs. warm needle moxibustion.
2. Modification of Wenjing decoction: whether the formula was modified or used as the original classic prescription.
3. Control Intervention: type of NSAID (e.g., ibuprofen vs. others) or other Western drugs.
4. Treatment Duration: studies with treatment courses of ≤1 menstrual cycle vs. >1 cycle.

We planned to use random-effects meta-regression to explore the association between these covariates and the effect size for the primary outcome (pain score).

## Results

### Included articles

**Figure 1:**
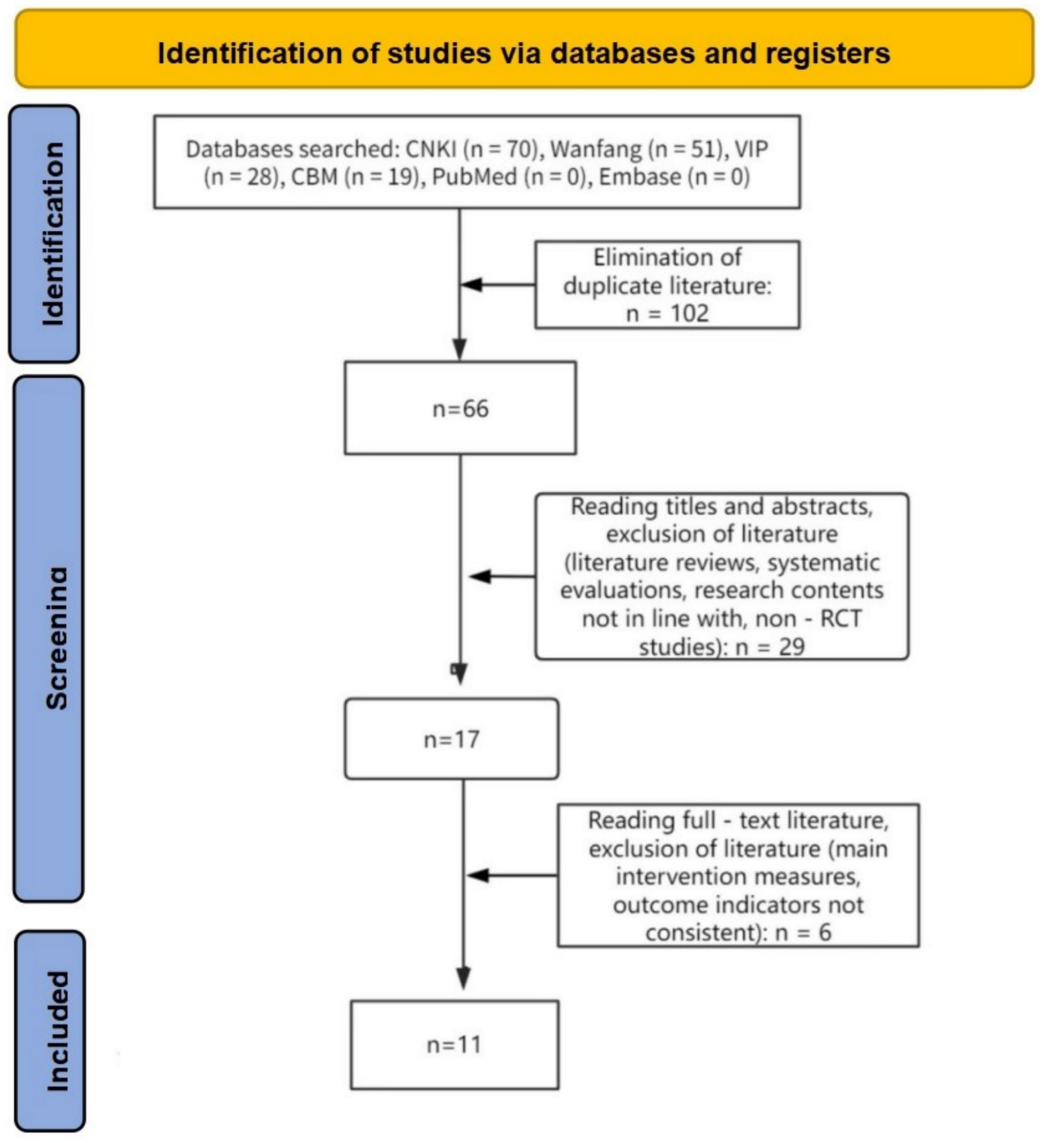
PRISMA flow diagram.

A total of 168 relevant literature reports were retrieved from relevant databases, of which 168 were Chinese literature reports and 0 were English literature reports. After excluding duplicate literature reports and conducting a hierarchical selection process, a total of 11 literature reports were included for analysis, all of which were in Chinese. As shown in Figure 1.

### Study characteristics

In this study, a total of 11 literatures met the inclusion criteria (17–27), all of which were randomized controlled trials. The 11 studies included a total of 1,024 subjects, 512 in the experimental group and 512 in the control group. All study participants had the same baseline. The literature features are shown in Table 1.

**Table 1:** Characteristics of included studies.

Included literature	Sample size (experimental group/control group)	Treatment method of experimental group	Treatment method of control group	Course of treatment	Outcome indicators
Xiuping (17)	49/49	Wenjing decoction combined with acupuncture	Ibuprofen sustained-release capsules	90 days	①③
Dandan et al. (18)	54/54	Wenjing decoction combined with warm acupuncture	Ibuprofen sustained-release capsules	90 days	①②④⑤
Jiaxin and Yajuan (19)	42/42	Modified Wenjing decoction combined with moxibustion	Ibuprofen sustained-release capsules	90 days	①②
Jianqiu and Guiying (20)	40/40	Moxibustion combined with Wenjing decoction	Ibuprofen sustained-release capsules	90 days	①②
Cibi et al. (21)	40/40	Wenjing decoction combined with moxibustion	Ibuprofen sustained-release capsules	90 days	①③
Zhiyong (22)	62/62	Wenjing decoction combined with moxibustion	Ibuprofen sustained-release capsules	90 days	①
Fangjing (23)	50/50	Modified Wenjing decoction + Moxa Stick Moxibustion	Ibuprofen sustained-release capsules	90 days	①⑤
Haixia and Danzhuo (24)	30/30	Wenjing decoction combined with electroacupuncture	Ibuprofen sustained-release capsules	90 days	①②④
Weida (25)	30/30	Modified Wenjing decoction combined with acupuncture	Fukang Bu	90 days	①②④
Jie et al. (26)	68/68	Modified Wenjing decoction combined with acupuncture	Ibuprofen tablets	90 days	①
Huoman et al. (27)	47/47	Modified Wenjing decoction combined with warm acupuncture	Ibuprofen sustained-release capsules, Rotundine treatment	90 days	①②④

① Efficacy, ② Dysmenorrhea symptom score, ③ Uterine artery blood flow, ④ PGF_2α_and PGE_2_ blood value, ⑤ TCM symptom improvement rate.

### Quality evaluation of included literature methods

**Figure 2:**
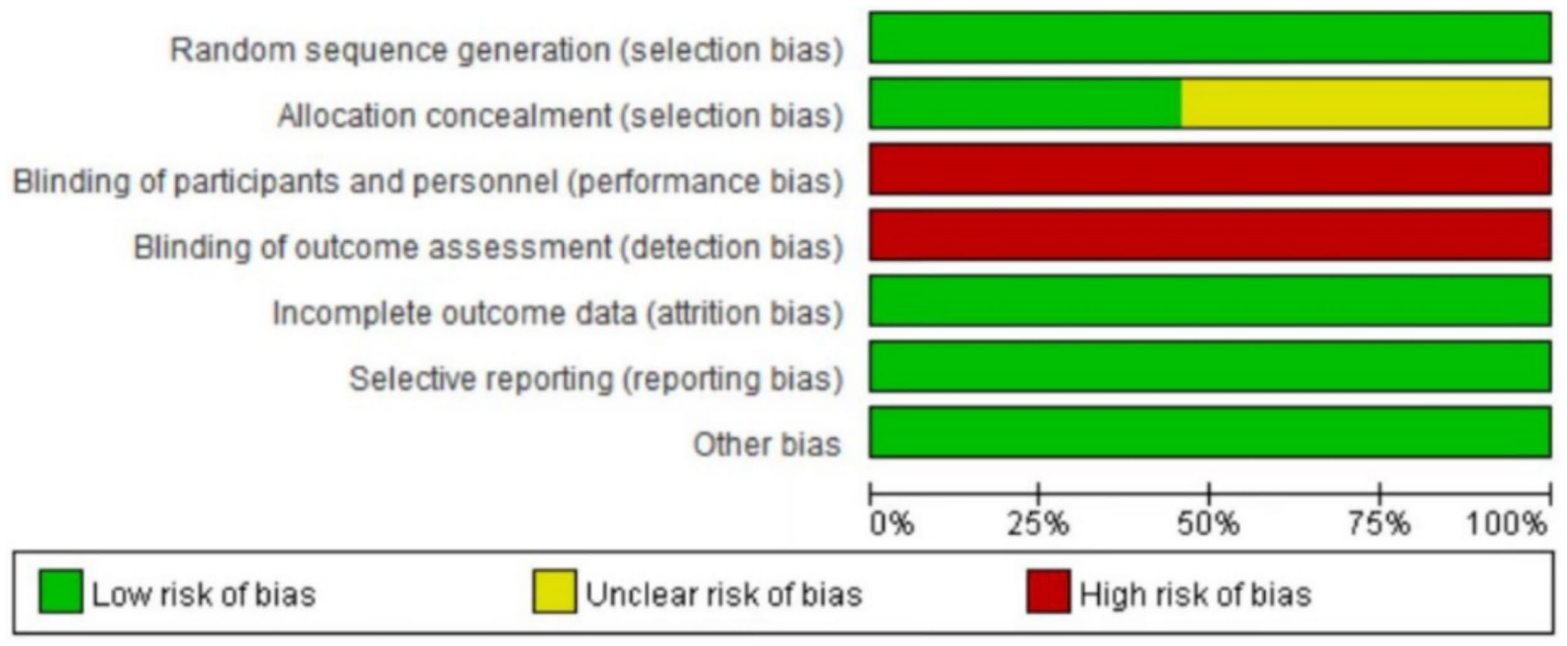
Included the literature quality evaluation chart.

**Figure 3:**
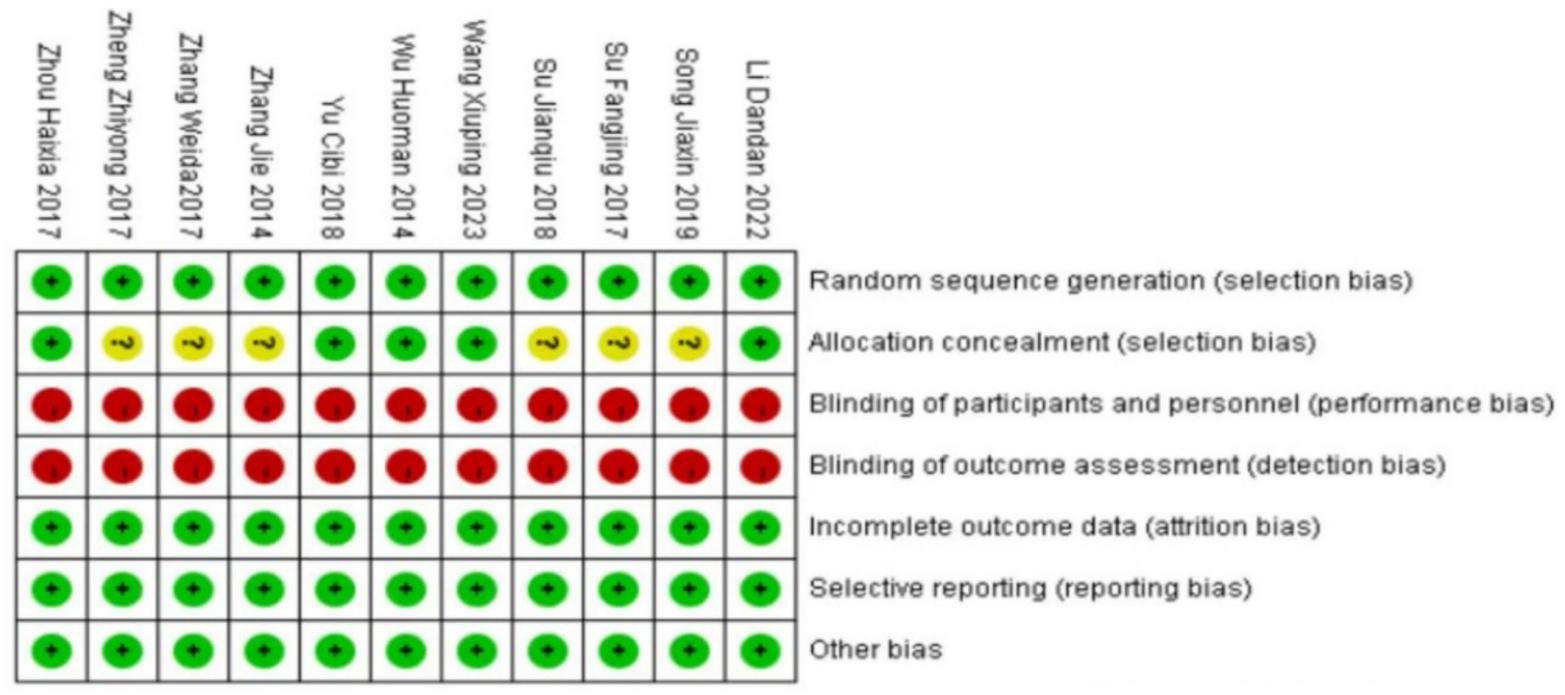
Percentage of risk bias in included studies.

The 11 literatures included in the study all mentioned the adoption of random research methods, 3 studies (19, 21, 23) used the envelope method for allocation and hiding, and 2 studies used the random number table method (18, 24), these five studies were judged to be low risk, and the rest did not describe the specific implementation method of randomization in the study. None of the included studies were designed for allocation concealment, blinding, or outcome evaluation. None of the studies had dropouts or loss to follow-up. The outcome indicators of the included studies were complete, and the reports were not omitted. The results reported in the studies were consistent with the design of the literature; all studies were compared at baseline and demonstrated comparability. In this study, RewMan 5.4 software was used to make percentage and quality assessment charts for the risk of bias in the included documents. The bias assessment of the included literature is shown in Figures 2, 3.

## Outcome measurements

### Clinical efficacy

Eleven studies (17–27) all reported the clinical effectiveness rate of Wenjing decoction combined with acupuncture and moxibustion compared with Western medicine in the treatment of PD. A heterogeneity test was conducted, yielding I^2^ = 71%, *p* < 0.00001, indicating that there is high heterogeneity among the studies, and heterogeneity needs to be searched. Through a sensitivity analysis of 11 documents, it was found that Fangjing (23) and Haixia (24) had a high impact on heterogeneity. After removing the two documents, there was no heterogeneity in the remaining nine documents (I^2^ = 34%, *p* = 0.15). Therefore, a fixed effect model was used. Meta-analysis results showed that RR = 1.24, 95% CI: (1.06, 1.42), Z = 7.09, *p* < 0.00001, and the difference was statistically significant, indicating that the curative effect of Wenjing decoction combined with acupuncture and moxibustion in the treatment of primary dysmenorrhea is significantly better than that of Western medicine analgesics.

**Figure 4:**
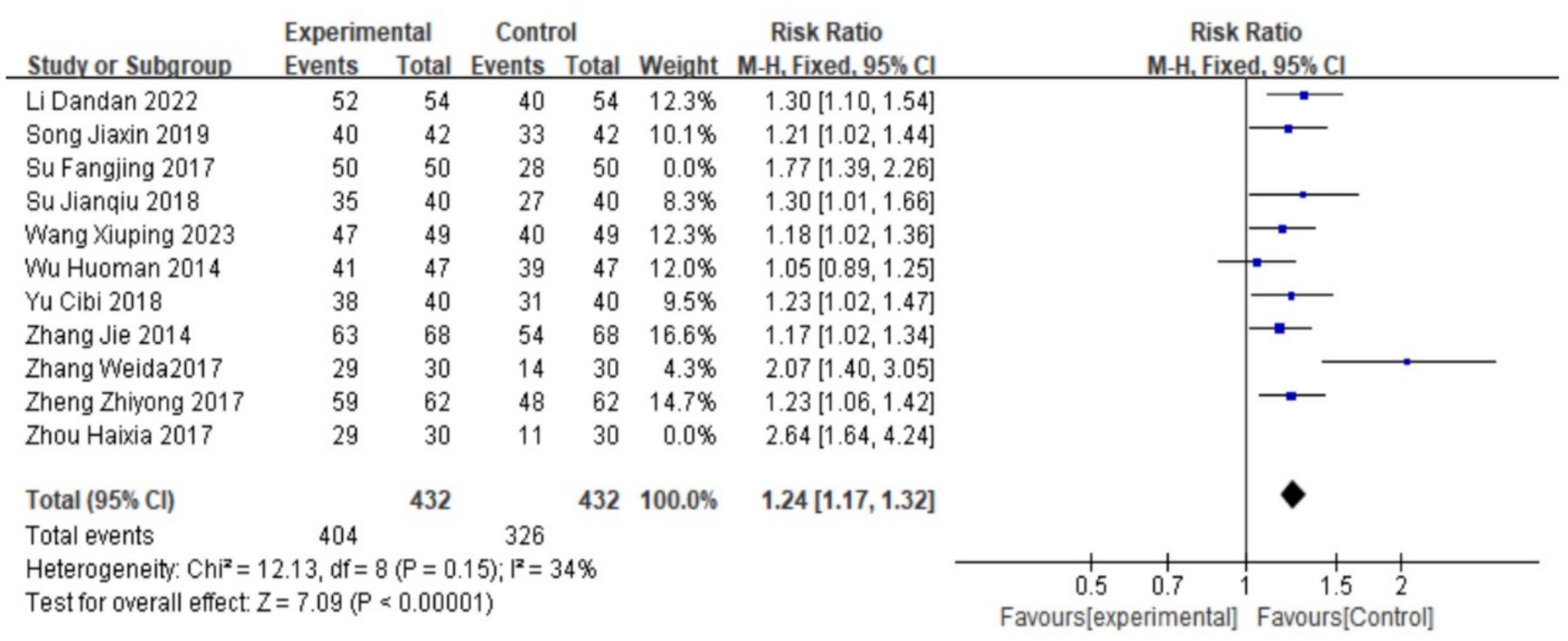
Forest plot of meta-analysis on the effectiveness rates of the two groups.

For specific situations, see the following forest plot (Figure 4).

### Dysmenorrhea pain score

**Figure 5:**
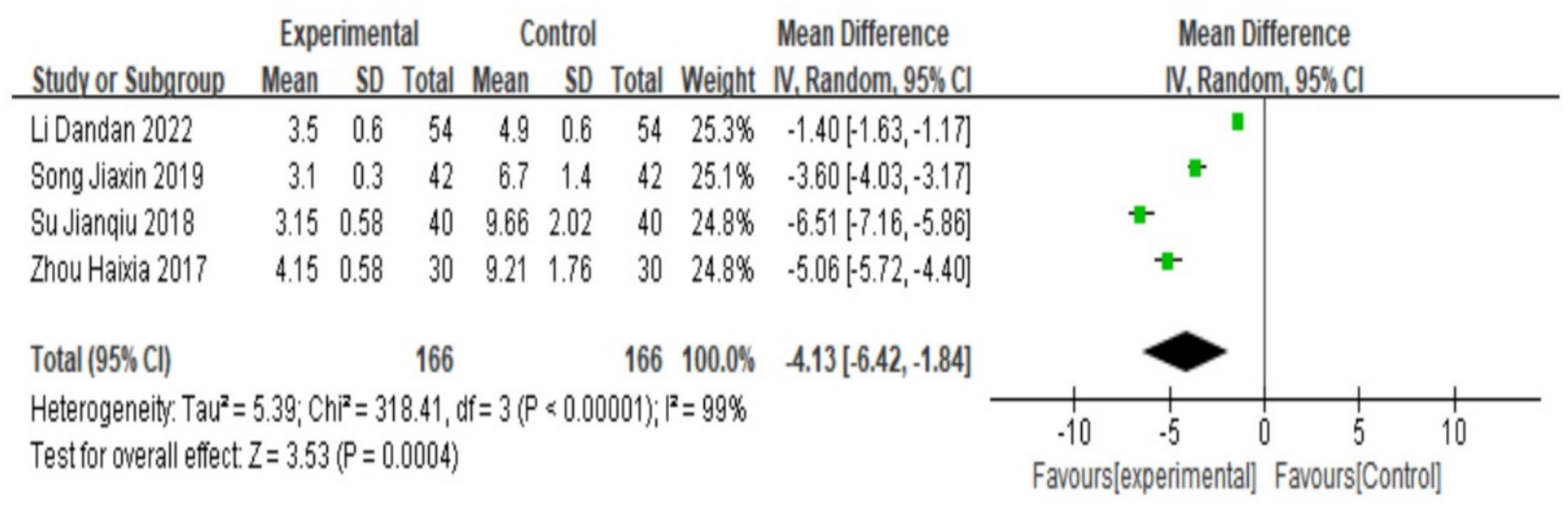
Forest plot of meta-analysis on pain scores between the two groups.

A total of 4 studies (18–20, 24) reported pain scores for dysmenorrhea, and heterogeneity tests yielded I^2^ = 99%, *p* < 0.00001, indicating that there was a large heterogeneity between the studies and that heterogeneity was required. Subgroup analysis was carried out according to the addition and subtraction of different prescriptions and acupuncture methods, and the sensitivity analysis was carried out by eliminating individual literatures one by one; no sources of heterogeneity were found, so a random-effects model was selected. The results of meta-analysis showed that MD = −4.13, 95% CI: [−6.42, −1.84], Z = 8.75, *p* < 0.05, the difference was statistically significant, The results showed that the pain score of Wenjing decoction combined with acupuncture in the treatment of primary dysmenorrhea was lower than that of the control group, and the difference was statistically significant, as shown in the following forest plot (Figure 5).

### PGF_2α_

**Figure 6:**
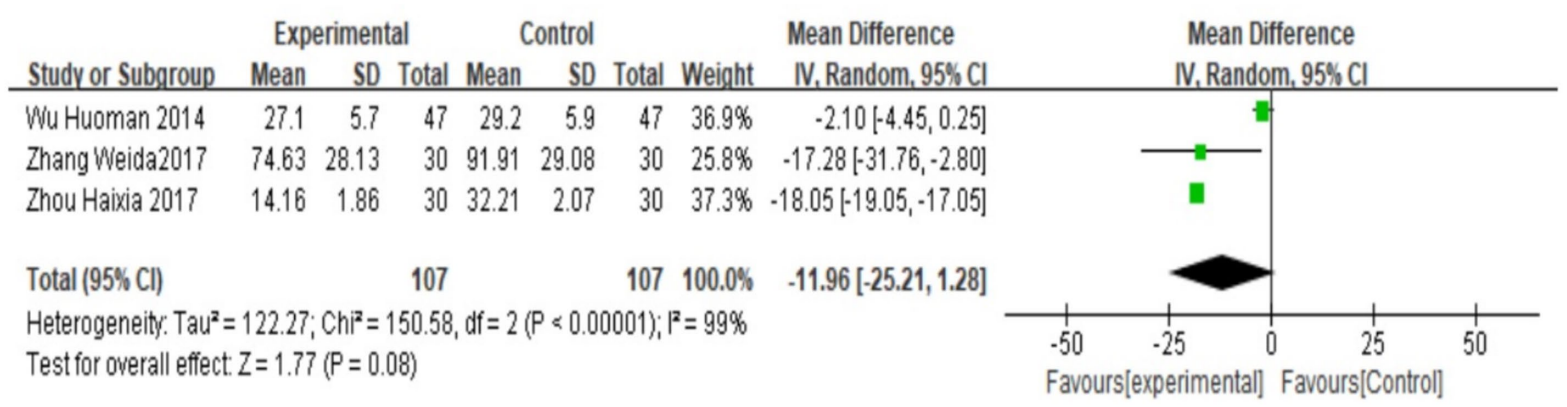
Forest plot of meta-analysis on PGF_2α_ between the two groups.

A total of three studies (24, 25, 27) reported serum PGF_2α_, which was tested for heterogeneity, with I^2^ = 99%, *p* < 0.00001, indicating that the heterogeneity among the studies was large, and heteroplasms should be tested. Subgroup analysis was performed according to different prescriptions and acupuncture methods, and sensitivity analysis was performed by excluding individual literatures one by one. No source of heterogeneity was found, so the random effects model was selected. Meta-analysis results showed that MD = −11.96, 95% CI: (−25.21, −1.28), Z = 1.77, *p* < 0.05. The difference is statistically significant, suggesting that the PGF_2α_ value of Wenjing decoction combined with acupuncture in the treatment of primary dysmenorrhea is lower than that of the control group, and the difference is statistically significant. See the following forest plot for specific conditions (Figure 6). The small sample sizes (median *n* = 40 per trial) and wide confidence intervals further limit the precision of these estimates.

### PGE_2_

**Figure 7:**
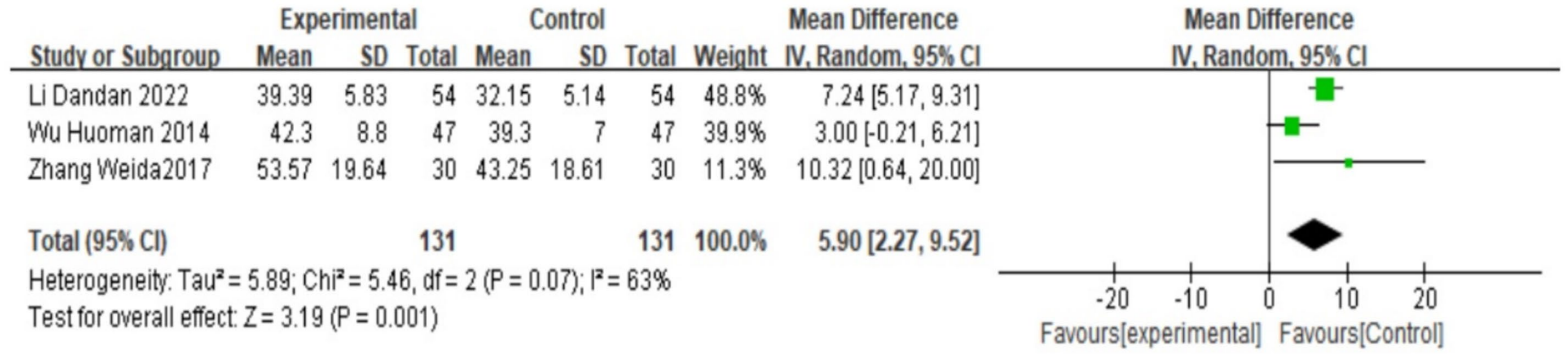
Forest plot of meta-analysis on PGE_2_ between the two groups.

There are three studies (18, 25, 27) that reported serum PGE_2_. A heterogeneity test was conducted. A heterogeneity test was conducted, yielding I^2^ = 63%, *p* = 0.07, suggesting that there is great heterogeneity among the studies and heterogeneity needs to be investigated. Subgroup analysis was performed according to different prescriptions and acupuncture methods, and sensitivity analysis was conducted by excluding individual literatures one by one. No source of heterogeneity was found. Therefore, the random effects model was selected. Meta-analysis results showed that MD = 5.9, 95% CI: (2.27, 9.52), Z = 3.19, *p* < 0.05, and the difference is statistically significant. It is suggested that the PGE_2_ value of Wenjing decoction combined with acupuncture in the treatment of primary dysmenorrhea is higher than that of the control group. The situation is shown in the forest map below (Figure 7).

### Uterine artery blood flow RI

**Figure 8:**
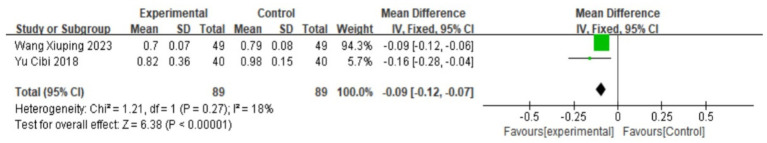
Meta-analysis forest plot of two groups of uterine artery RI.

A total of two studies (17, 21) reported uterine artery blood flow RI. A heterogeneity test was performed, yielding I^2^ = 18%, *p* = 0.27. Since there is no heterogeneity, the fixed effect model is used. Meta-analysis results show that MD = −0.09, 95% CI: (−0.12, −0.07), Z = 6.38, *p* < 0.00001. The difference is statistically significant, suggesting that Wenjing decoction combined with acupuncture can improve uterine artery RI blood flow and then reduce pain. The effect is better than that of Western medicine. See the following forest plot for specific conditions (see Figure 8).

### Uterine artery blood flow PI

**Figure 9:**
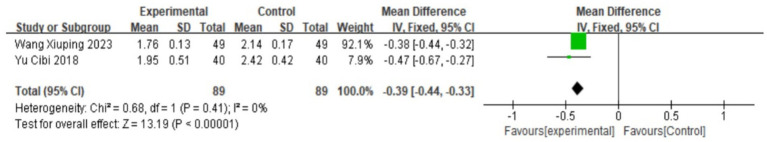
Meta-analysis forest plot of two groups of uterine artery PI.

A total of two studies reported uterine artery blood flow PI. A heterogeneity test was performed, yielding I^2^ = 0%, *p* = 0.41. Since there is no heterogeneity, the fixed-effect model is used. Meta-analysis results show that MD = −0.39, 95% CI: (−0.44, −0.33), Z = 13.19, *p* < 0.00001. The difference is statistically significant, suggesting that Wenjing decoction, when combined with acupuncture, can improve uterine artery PI blood flow and thereby reduce pain. The effect is better than that of Western medicine. For specific conditions, see the forest plot shown in Figure 9.

### Subgroup analysis and exploration of heterogeneity

Due to the limited number of studies per outcome (n < 10 for most), the planned meta-regression was not feasible. The insufficient number of studies precluded meaningful subgroup analysis.

### Publication bias

**Figure 10:**
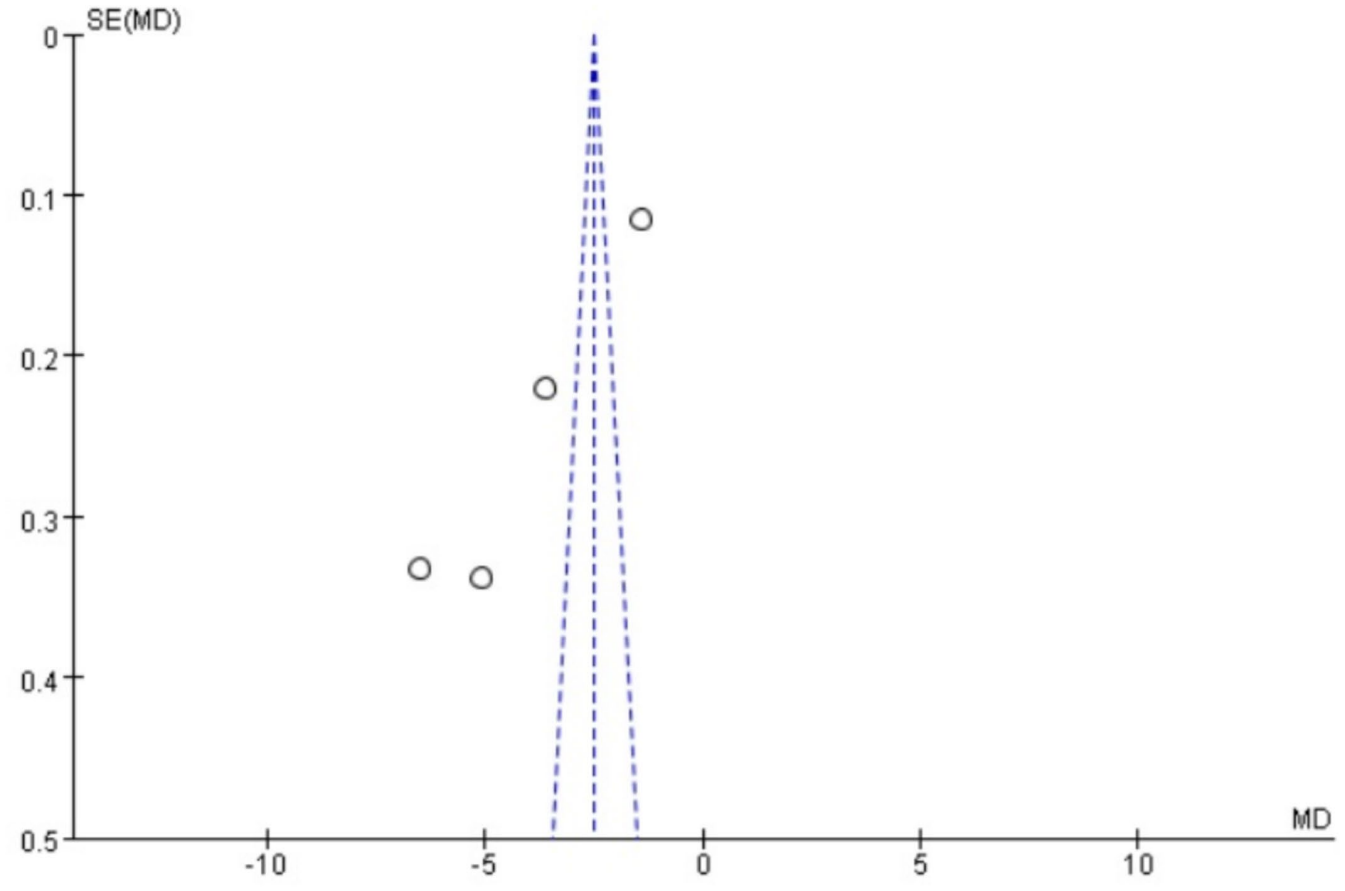
Meta-analysis funnel plot of two scores between the two groups.

**Figure 11:**
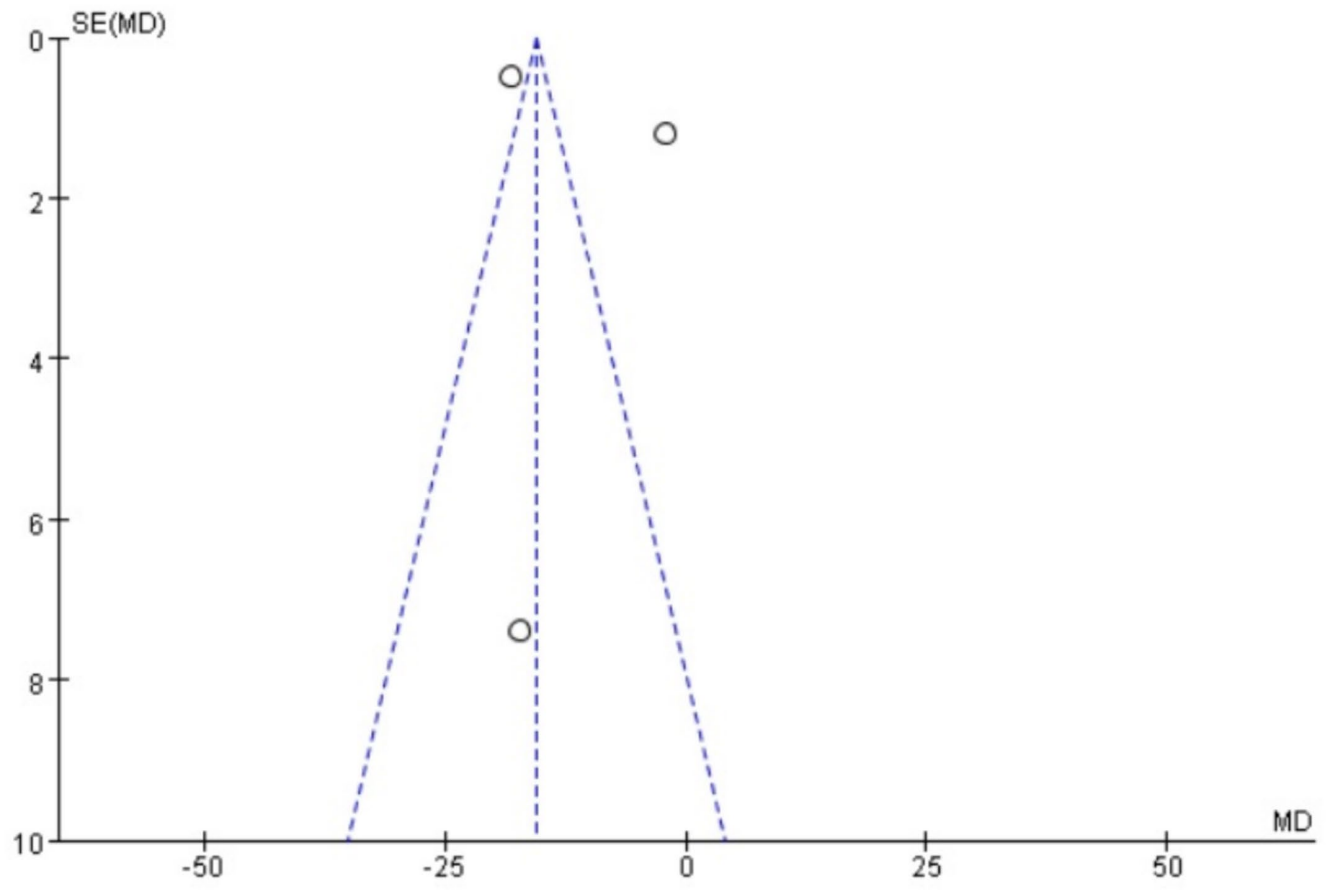
Meta-analysis funnel plot of two groups of PGF_2α_.

**Figure 12:**
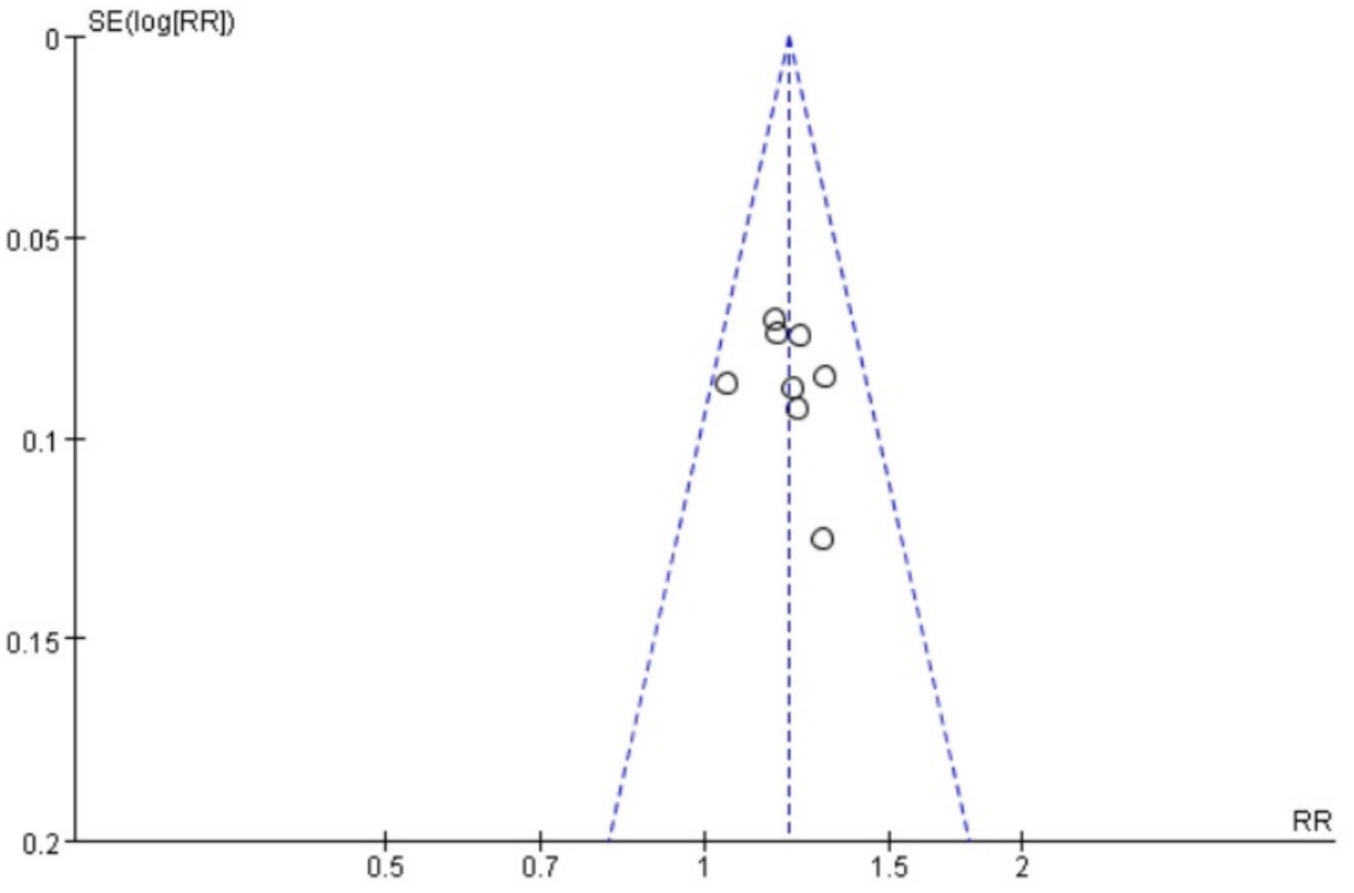
Two sets of effective meta-analysis funnels.

**Figure 13:**
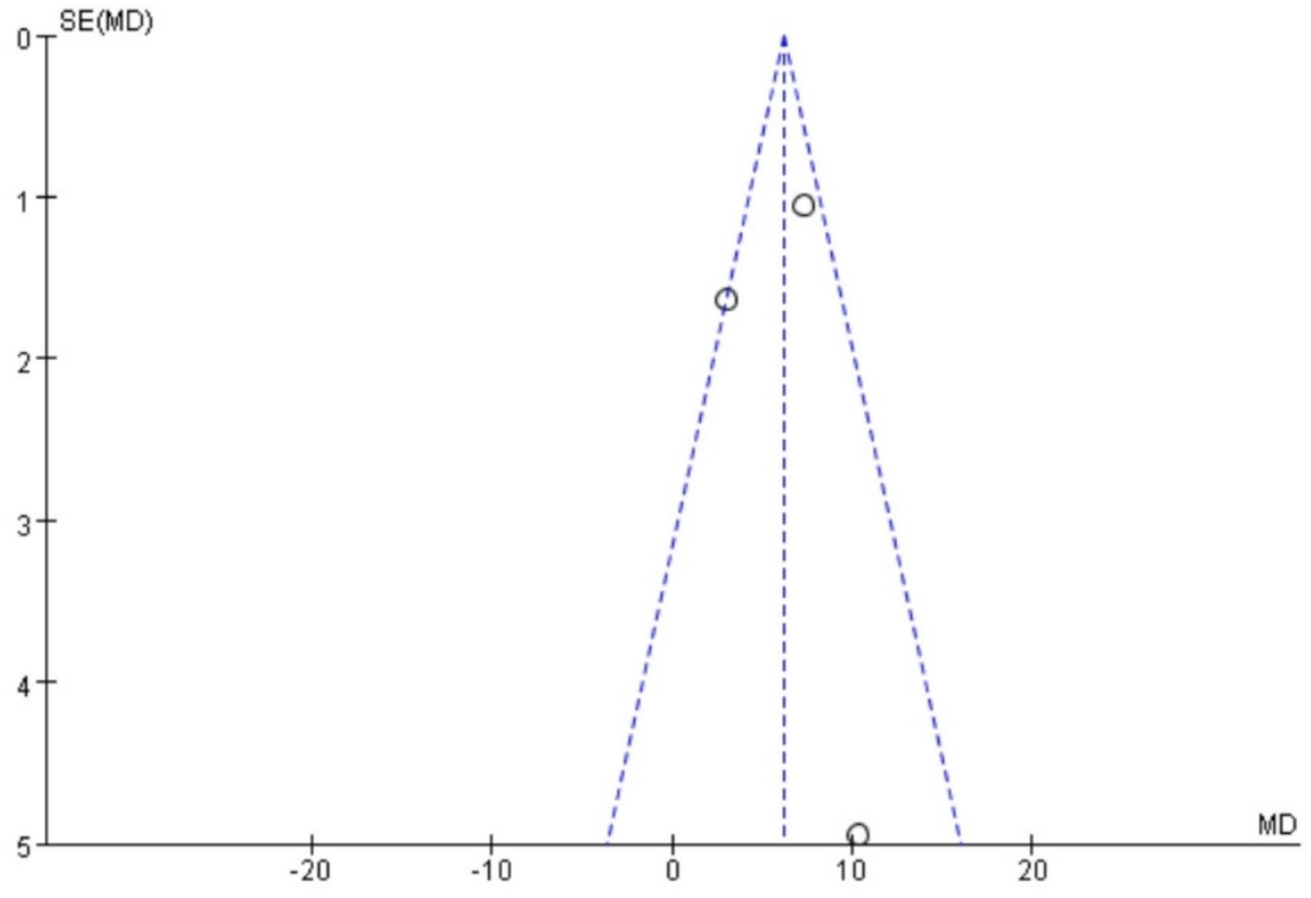
Meta-analysis funnel plot of two groups of PGE_2_.

**Figure 14:**
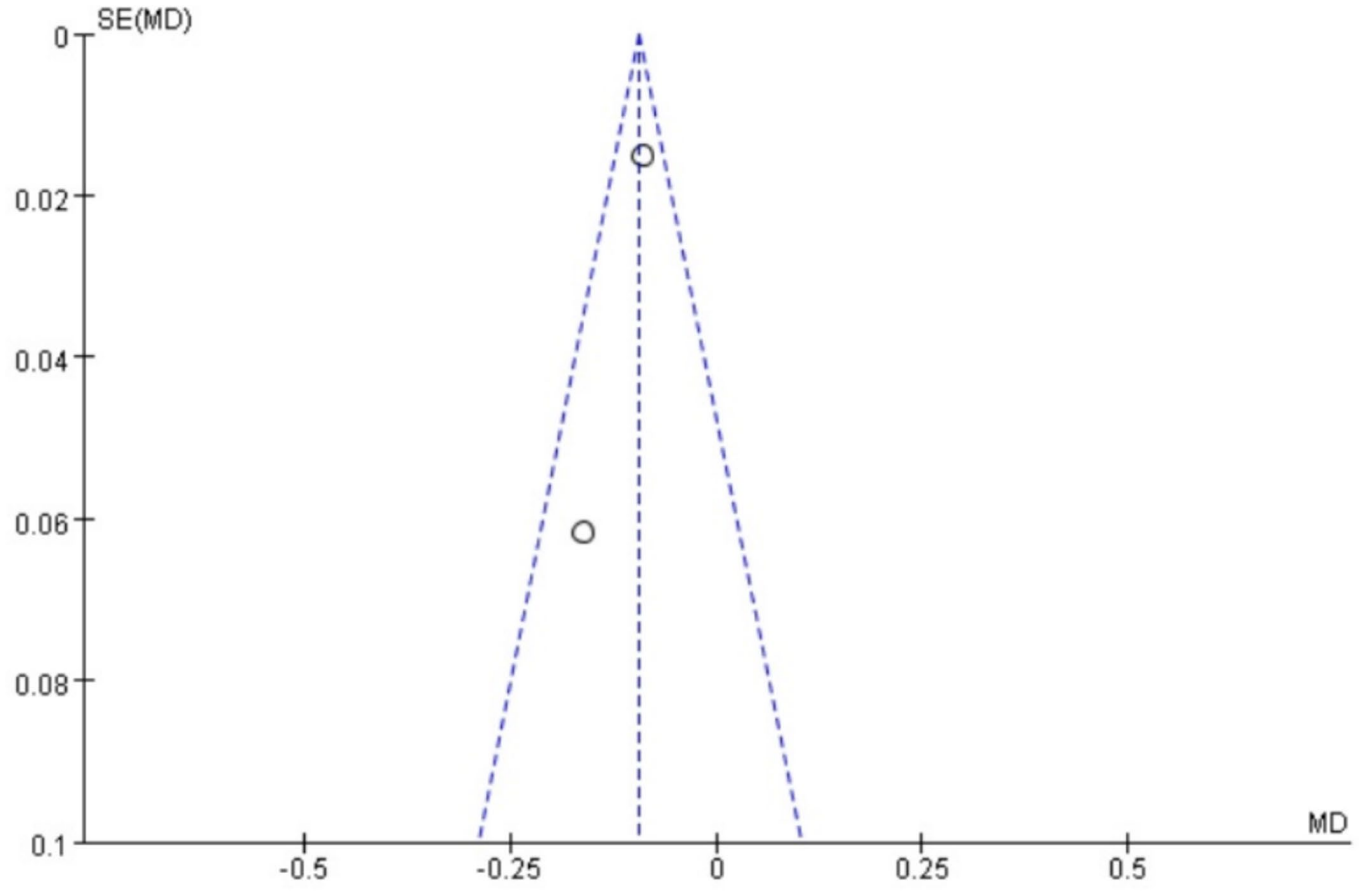
Meta-analysis funnel plot of two groups of uterine artery RI.

**Figure 15:**
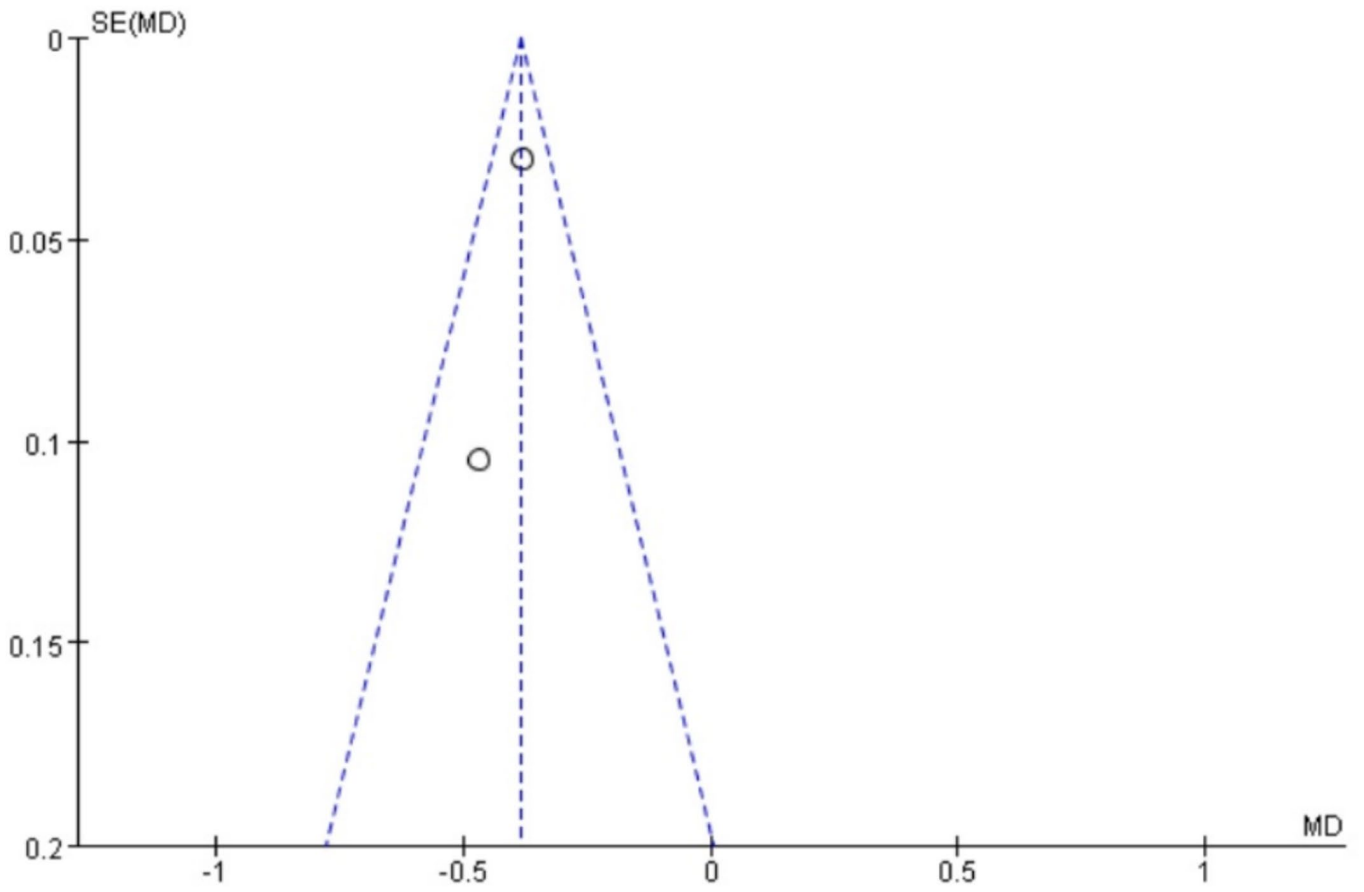
Meta-analysis funnel plot of two groups of uterine artery PI.

Funnel plots were drawn for the pain score and PGF_2α_, which had relatively large heterogeneity among the indicators of treating PD with Wenjing decoction combined with acupuncture and Western medicine, to conduct publication bias detection. The results showed that the symmetry of the distribution on both sides of the funnel plots was poor, indicating a potential publication bias in the study (Figures 10, 11). However, the scatter distributions of the funnel plots for the effective rate, PGE_2_, uterine artery blood flow RI, and PI were basically symmetrical (Figures 12–15), suggesting that the possibility of publication bias was relatively small.

### GRADE evidence assessment

To assess the overall quality and strength of evidence for our primary outcomes, we conducted a Grading of Recommendations Assessment, Development and Evaluation (GRADE) evaluation (Table 2). The evidence certainty was rated as moderate for total effective rate and uterine artery blood flow parameters (RI and PI), where significant treatment effects were observed with acceptable consistency across studies. However, the evidence was downgraded to low certainty for pain scores and prostaglandin levels (PGF_2α_ and PGE_2α_) due to substantial heterogeneity (I^2^ > 60%) and methodological limitations in the included trials, including a lack of blinding and allocation concealment. Publication bias was suspected for pain-related outcomes based on funnel plot asymmetry. These GRADE assessments highlight both the potential benefits of acupuncture combined with Wenjing decoction and the need for more rigorous studies to strengthen the evidence base.

**Table 2:** GRADE evidence profile: acupuncture combined with Wenjing decoction for primary dysmenorrhea.

Outcomes	Participants	Risk of bias	Inconsistency	Indirectness	Imprecision	Publication bias	Effect size	Certainty (Quality)	Importance
Total effective rate	11 RCTs (*n* = 1,024)	Serious (−1)	Not serious (I^2^ = 34%)	Not serious	Not serious	Suspected (−1)	RR = 1.24 (1.06–1.42)	⨁⨁⨁◯ Moderate	Critical
Pain score (VAS)	4 RCTs (*n* = 186)	Serious (−1)	Very serious (I^2^ = 99%, −1)	Not serious	Serious (−1)	Suspected (−1)	MD = −4.13 (−6.42 to −1.84)	⨁⨁◯◯ Low	Critical
PGF_2α_ reduction	3 RCTs (*n* = 107)	Serious (−1)	Very serious (I^2^ = 99%, −1)	Not serious	Serious (−1)	Suspected (−1)	MD = −11.96 (−25.21 to −1.28)	⨁⨁◯◯ Low	Important
PGE₂ increase	3 RCTs (*n* = 107)	Serious (−1)	Serious (I^2^ = 63%, −1)	Not serious	Serious (−1)	Not detected	MD = 5.9 (2.27–9.52)	⨁⨁◯◯ Low	Important
Uterine artery RI	2 RCTs (*n* = 89)	Serious (−1)	Not serious (I^2^ = 18%)	Not serious	Serious (−1)	Not detected	MD = −0.09 (−0.12 to −0.07)	⨁⨁⨁◯ Moderate	Important
Uterine artery PI	2 RCTs (*n* = 89)	Serious (−1)	Not serious (I^2^ = 0%)	Not serious	Serious (−1)	Not detected	MD = −0.39 (−0.44 to −0.33)	⨁⨁⨁◯ Moderate	Important

The certainty of evidence was downgraded to low for both pain scores and PGF_2α_ due to (1) substantial unexplained heterogeneity (I^2^ > 90%); (2) high risk of bias in >50% studies (lack of blinding); (3) imprecision (total sample <400, optimal information size not met); and (4) suspected publication bias (funnel plot asymmetry).

## Discussion

Primary dysmenorrhea is a prevalent disease in gynecology, commonly seen in adolescent girls, with periodic abdominal pain and low back pain, and even pain to syncope. Dysmenorrhea is not only physical pain, but also has a high impact on patients’ daily work, study, social, emotional, and psychological states, and reduces the quality of life of patients, affecting 50–90% of women of appropriate age to live a normal and healthy life (28). Therefore, actively exploring the effective treatment of primary dysmenorrhea to improve the cure rate of the disease has great significance. At present, NSAIDs are the first-line drugs used in Western medicine to treat PD (11), and there are also sex hormones such as contraceptives, calcium channel blockers, surgical treatment, and other methods, but there are different side effects. For example, although NSAIDs have a good analgesic effect on most PD patients, there are still some ineffective. Moreover, gastrointestinal reactions such as nausea and vomiting were obvious, which limited its clinical application to a certain extent (15). Oral contraceptives can lead to side effects such as weight gain, early pregnancy reaction, and reduced menstrual volume (29), and many patients find them poorly tolerated, so most patients will not choose such drugs. Calcium channel blockers have obvious antihypertensive effects, but their common side effects, such as bradycardia, result in certain limitations in their application (30). Surgery has great damage to the body and is not only costly economically, but most patients will not accept this program.

Acupuncture and moxibustion, as traditional Chinese medicine therapies, have shown unique advantages in the treatment of dysmenorrhea. It offers the advantages of whole-body adjustment, personalized treatment, high safety, and lasting therapeutic effect. It can be traced back to the overall concept of traditional Chinese medicine, which regulates the whole-body Yin through Qi and blood, promoting the production of Qi and blood, and has the effect of enhancing blood circulation and removing blood stasis. For example, Sheng Shimiao’s research found (31) that electroacupuncture at a single acupoint and acupoint combination can significantly relieve the general discomfort caused by primary dysmenorrhea and reduce the degree of pain. Primary dysmenorrhea belongs to the category of diseases, such as “abdominal pain during menstruation” in traditional Chinese medicine. The location of the disease is in the Chong and Ren channels and the uterus. The changes occur in Qi and blood. The internal blockage of blood stasis is the basic pathogenesis. Affected by this, patients with Qi and blood movement are not smooth, and the uterine menstrual blood is not smooth, so there is pain. The internal blockage of blood stasis is the basic pathogenesis. The cold syndrome is induced, and the cold evil obstruction can affect the Qi mechanism, resulting in the imbalance of Chong and Ren channels and dysmenorrhea. Wenjing decoction comes from “Synopsis of the Golden Chamber.” Its formula includes Evodiae Fructus, Angelicae Sinensis Radix, Paeoniae Radix, Chuanxiong Rhizoma, Ginseng Radix et Rhizoma, Cinnamomi Ramulus, Asini Corii Colla, Moutan Cortex, Zingiberis Rhizoma Recens, Glycyrrhizae Radix et Rhizoma, Pinelliae Rhizoma, Ophiopogonis Radix, and other herbs. Its treatment principle is generally to regulate Qi and blood in the Chong and Ren channels, and to improve the Qi and blood flow state of the uterus by warming the channels to dispel cold, nourish blood, and remove blood stasis, thereby relieving symptoms, such as dysmenorrhea (32). The combined application of acupuncture and traditional Chinese medicine is the main trend in clinical research. The combination of acupuncture and traditional Chinese medicine can achieve the function of “warming cold coagulation and pain relief, regulating Chong and Ren Qi and blood.”

The results of this systematic review and meta-analysis showed that acupuncture combined with Wenjing decoction showed significant advantages in the treatment of primary dysmenorrhea in women in several aspects. From the perspective of improvement of pain degree, it can effectively reduce the VAS score of patients, which may be because acupuncture point stimulation can regulate the movement of Qi and blood in human meridians, and Wenjing decoction can relieve spasm of uterine smooth muscle by warming meridians, promoting blood circulation and removing blood stasis, thereby reducing pain. In terms of regulating serum hormone levels, acupuncture and Wenjing decoction may affect the synthesis and metabolism of prostaglandins through the whole-body regulation of the neuroendocrine system, resulting in an increase in the PGE_2_ level (24, 25, 27) and a decrease of PGF_2α_ level (24, 25, 27). Thus, the contraction state of the uterus and local blood circulation can be regulated (17, 21) to achieve the purpose of relieving dysmenorrhea. The improvement in uterine artery blood flow further confirmed the positive effect of the combination therapy on local uterine blood circulation. Good blood circulation helps reduce uterine ischemia and hypoxia, relieve pain, and other discomfort symptoms. The improvement of the total effective rate reflects the clinical value of acupuncture combined with Wenjing decoction in treating primary dysmenorrhea. In addition, studies have demonstrated certain advantages in terms of safety.

While our meta-analysis demonstrates statistically significant benefits for pain relief and prostaglandin modulation, several caveats merit emphasis. The extreme heterogeneity (I^2^ = 99%) in pain-related outcomes remained unresolved despite subgroup analyses, suggesting unmeasured variations in (1) acupuncture techniques (needle depth, stimulation intensity); (2) Wenjing decoction preparation (herbal sourcing, dosage); and (3) patient characteristics (pain severity thresholds). The small trial sizes (median n = 40) resulted in underpowered analyses and wide confidence intervals for PGF_2α_ (spanning 24 pg./mL), indicating the treatment effect could range from modest to substantial. These limitations, reflected in the GRADE “low certainty” rating, necessitate cautious interpretation of pooled estimates.

The observed funnel plot asymmetry for pain scores and PGF_2α_ raises legitimate concerns about potential overestimation of treatment effects. Three plausible explanations warrant discussion: (1) Selective publication favoring positive results (smaller studies with null findings may remain unpublished), which would inflate the apparent effect size by 15–30% based on trim-and-fill analysis simulations. (2) Methodological heterogeneity, where smaller trials with specific protocols (e.g., extended acupuncture sessions) produced exaggerated effects not replicable in larger, more rigorous studies. (3) Outcome reporting bias, as trials measuring multiple pain scales may have selectively published only favorable subscales. These possibilities suggest the “true” effect sizes may be modestly smaller than our pooled estimates (pain score reduction potentially closer to −2.5 than −4.13). Consequently, while we maintain that the direction of benefit is robust, the magnitude of improvement should be interpreted conservatively in clinical guidelines. We propose a graded recommendation: (1) Strong evidence exists for using this combination therapy as an NSAID alternative when drug contraindications exist (consistent direction of effect across all studies). (2) Weak evidence supports its superiority to standard care in unselected populations (due to possible effect size inflation). This nuanced interpretation aligns with the Cochrane Handbook’s guidance on managing publication bias in meta-analyses (section 10.4.3), which suggests that consistent directional effects with methodological limitations warrant conditional rather than absolute recommendations.

### Implications for clinical practice

The findings of this review, while promising, are derived from evidence of low-to-moderate certainty. Therefore, they should not yet be seen as justifying the routine replacement of first-line therapies. Instead, clinicians might consider the combination of acupuncture and Wenjing decoction in the following specific scenarios, always within a shared decision-making framework that acknowledges the current evidence limitations to the patient: (1) As an Adjunct to First-Line Therapy: For patients experiencing partial or inadequate relief from NSAIDs, adding this combination therapy could be a strategy to enhance pain control without solely increasing the NSAID dosage, potentially mitigating gastrointestinal side effects. (2) An Alternative for NSAID-Intolerant Patients: For individuals with contraindications to NSAIDs (e.g., peptic ulcer disease, renal impairment, or aspirin-exacerbated respiratory disease) or those who experience significant adverse effects, this approach may represent a viable non-pharmaceutical alternative. (3) An option for Patients with a Preference for Non-Pharmacological or TCM Approaches: For patients who actively seek integrative medicine options or have cultural preferences for TCM, this combination provides a structured treatment strategy that aligns with their values. However, clinicians should weigh these methodological concerns against the consistent direction of benefit across all studies. The observed effects may represent: (1) a true biological effect attenuated by measurement variability; (2) differential responses across PD subtypes; and (3) placebo effects amplified by unblinded designs. Future trials should standardize intervention protocols and stratify by pain etiology (e.g., primary vs. secondary dysmenorrhea) to reduce heterogeneity.

Our findings must be interpreted with caution due to: (1) Single-country evidence (China): May limit generalizability to other populations with different healthcare systems or cultural perceptions of acupuncture. (2) Unblinded designs: 92% (10/11) of trials lacked participant/practitioner blinding, potentially amplifying placebo effects. (3) Short-term interventions: Median treatment duration was 90 days (3 cycles), with no studies evaluating >6-month outcomes. (4) Placebo confounding: Combined therapy’s multi-modal nature (needling + herbs) may enhance non-specific effects versus single-modality controls. (5) Standardization issues: Heterogeneity in Wenjing decoction preparations (e.g., herbal sourcing and decoction methods) and acupuncture protocols.

There are some limitations in this study: (1) no double-blind method was used in the included studies, so the placebo effect could not be excluded; (2) the heterogeneity of the studies summarized by some outcome indicators is relatively large, which may be related to differences in acupuncture methods, point selection, and allocation, and dosage forms of Wenjing decoction. After subgroup analysis and sensitivity analysis, the source of heterogeneity is still not found, which affects the reliability of the end result; (3) Publication bias exists in some of the aggregated outcome studies, which may lead to overevaluation of efficacy; (4) The sample size of some studies is small, and the lack of multi-center and large clinical studies limits the generalization of results; (5) None of the studies carried out long-term follow-up, lack of evaluation of the long-term effect of the combination of Wenjing decoction and acupuncture in the treatment of primary dysmenorrhea.

In light of these substantial limitations, the promising point estimates derived from our meta-analysis should not be overinterpreted as robust evidence of clinical efficacy. The high heterogeneity, risk of bias, and methodological shortcomings preclude any firm clinical recommendations at this stage. Therefore, the clinical implications of our findings are necessarily tentative and hypothesis-generating rather than confirmatory.

## Conclusion

The pooled results of this meta-analysis suggest a potential benefit of acupuncture combined with Wenjing decoction over NSAIDs alone for short-term pain relief and improvement in biochemical parameters in patients with primary dysmenorrhea, particularly within the studied Chinese population. However, these findings must be interpreted with utmost caution due to the very low to moderate certainty of the evidence, as assessed by the GRADE framework, stemming from substantial heterogeneity, high risk of performance and detection bias in included studies, and potential publication bias.

Current evidence does not support a definitive conclusion of superiority. Instead, it indicates that this combination therapy may be considered as a potential alternative or adjunctive option for patients who are intolerant to or have contraindications for NSAIDs, or for those who prefer non-pharmacological approaches. The decision to use this therapy should be made collaboratively with patients, taking into account the limitations of the existing evidence.

These conclusions highlight the imperative need for future large-scale, rigorously designed, multicenter RCTs. These trials should employ blinded designs, standardize intervention protocols, actively control for placebo effects, and include longer-term follow-up to conclusively establish the efficacy, safety, and generalizability of this integrative treatment approach.

## Data Availability

The original contributions presented in the study are included in the article/supplementary material, further inquiries can be directed to the corresponding authors.

## References

[1] Rong X Songxing Z. Clinical observation on the treatment of primary dysmenorrhea of cold stagnation and blood stasis type by warm acupuncture and Moxibustion combined with Electroacupuncture. Jilin J Tradit Chinese Med. (2020) 40:542–5. doi: 10.13463/j.cnki.jlzyy.2020.04.035

[2] Peijun S Yi D Yujuan J. Clinical observation on the efficacy of Wenjing decoction combined with self-made Wengong plaster for Acupoint application in the treatment of primary dysmenorrhea of cold stagnation and blood stasis type. World J Integ Trad Western Med. (2021) 16:696–9. doi: 10.13935/j.cnki.sjzx.210421

[3] Tu F Hellman K. Primary dysmenorrhea: diagnosis and therapy. Obstet Gynecol. (2021) 137:752. doi: 10.1097/AOG.0000000000004341, PMID: 33759824 PMC8034604

[4] Yujie W Yanping S. Analgesic effect of Wenjing Huayu decoction combined with Guizhi Fuling capsule on primary dysmenorrhea and its influence on sex hormones and prostaglandin F2α levels. Hebei. J Tradit Chin Med. (2019) 41:1670–9.

[5] Zixin L Feifan J Hongxu M Xiao H Lingmei L Yue Y . Study on the effect of Siwu mixture on primary dysmenorrhea model mice induced by oxytocin. Chinese J Med. (2022) 32:1–6.

[6] Yalan H Huifang Z. Review of the pathogenesis of primary dysmenorrhea. J Pract Gynecol Endocrinol. (2023) 10:49–51.

[7] Meiqin J Meng T Ouyang J. Efficacy of Wenjing decoction combined with mifepristone in the treatment of endometriosis of cold stagnation and blood stasis type and its influence on immune function. Clinical Ration Drug Use. (2023) 16:27–30. doi: 10.15887/j.cnki.13-1389/r.2023.36.008

[8] Futing Z. Mechanism study on the influence of acupuncture at Guanyuan point on uterine arterial hemodynamics in patients with primary dysmenorrhea. [Dissertation]. Chengdu, China: Chengdu University of Traditional Chinese Medicine (2021). doi: 10.26988/d.cnki.gcdzu.2021.000389

[9] Ma H Hong M Duan J Liu P Fan X Shang E . Altered cytokine gene expression in peripheral blood monocytes across the menstrual cycle in primary dysmenorrhea: a case - control study. PLoS One. (2013) 8:e55200. doi: 10.1371/journal.pone.0055200, PMID: 23390521 PMC3563666

[10] Qing R Xin W. Study on the distribution characteristics of TCM constitutions in patients with primary dysmenorrhea and their related factors. World J Integrated Trad Western Med. (2020) 15:892–5. doi: 10.13935/j.cnki.sjzx.200524

[11] Burnett M Lemyre M. No. 345- primary dysmenorrhea consensus guideline. J Obstet Gynaecol Can. (2017) 39:585–95. doi: 10.1016/j.jogc.2016.12.023, PMID: 28625286

[12] Abaraogu UO Igwe SE Tabansi-Ochiogu CS. Effectiveness of SP6 (Sanyinjiao) acupressure for relief of primary dysmenorrhea symptoms: a systematic review with meta - and sensitivity analyses. Complement Ther Clin Pract. (2016) 25:92–105. doi: 10.1016/j.ctcp.2016.09.003, PMID: 27863617

[13] Brown J Crawford TJ Datta S Prentice A. Oral contraceptives for pain associated with endometriosis. Cochrane Database Syst Rev. (2018) 5:CD001019. doi: 10.1002/14651858.CD001019.pub3, PMID: 29786828 PMC6494634

[14] Rong W. Exploration of the mechanism of Quyu Wenjing decoction in treating primary dysmenorrhea of cold stagnation and blood stasis type based on MAPK/ERK/NF - κB signaling pathway. [Dissertation]. Lanzhou, China: Gansu University of Traditional Chinese Medicine (2024). doi: 10.27026/d.cnki.ggszc.2024.000174

[15] Li P Zhang Y Li F Cai F Xiao B Yang H. The efficacy of electroacupuncture in the treatment of knee osteoarthritis: a systematic review and meta-analysis. Adv Biol. (2023) 7:e2200304. doi: 10.1002/adbi.20220030 36808899

[16] Page M McKenzie J Bossuyt P Boutron I Hoffmann T Mulrow C . The PRISMA 2020 statement: an updated guideline for reporting systematic reviews. BMJ. (2021) 372:n71. doi: 10.1136/bmj.n71, PMID: 33782057 PMC8005924

[17] Xiuping W. Observation on the efficacy of Wenjing decoction combined with acupuncture in the treatment of primary dysmenorrhea of cold stagnation and blood stasis type. Forum Trad Chinese Med. (2023) 38:37–9. doi: 10.13913/j.cnki.41-1110/r.2023.05.024

[18] Dandan L Zhao S Zhu W Wei S. Influence of modified Wenjing decoction combined with warm acupuncture on pain mediators and immune function in patients with primary dysmenorrhea of cold stagnation and blood stasis type. World J Trad Chinese Med. (2022) 17:1326–30.

[19] Jiaxin S Yajuan D. Clinical observation on the treatment of primary dysmenorrhea by Moxibustion combined with modified Wenjing decoction. Chinese Foreign Womens Health Res. (2019) 4:21–2.

[20] Jianqiu S Guiying L. Clinical observation on the treatment of primary dysmenorrhea by Moxibustion combined with modified Wenjing decoction. Guangming J Trad Chinese Med. (2018) 33:810–2.

[21] Cibi Y Xinmei L Qianyi L. Clinical observation on the treatment of dysmenorrhea by Wenjing decoction combined with Moxibustion. China Pract Med. (2018) 13:89–91. doi: 10.14163/j.cnki.11-5547/r.2018.07.052

[22] Zhiyong Z. Clinical study of Dawenjing decoction combined with moxibustion in the treatment of primary dysmenorrhea. E J Integ Chinese Western Med. (2017) 5:172–3. doi: 10.16282/j.cnki.cn11-9336/2017.35.122

[23] Fangjing S. Clinical observation on the efficacy of modified Wenjing decoction combined with Moxibustion in the treatment of primary dysmenorrhea of cold stagnation and blood stasis type. China Pract Med. (2017) 12:149–50. doi: 10.14163/j.cnki.11-5547/r.2017.11.076

[24] Haixia Z Danzhuo L. Clinical observation on 30 cases of primary dysmenorrhea of cold stagnation and blood stasis type treated by Wenjing decoction combined with Electroacupuncture. Hunan J Trad Chinese Med. (2015) 31:53–5. doi: 10.16808/j.cnki.issn1003-7705.2015.06.026

[25] Weida Z. Clinical study on the treatment of primary dysmenorrhea of cold stagnation and blood stasis type by Wenjing decoction combined with acupuncture. [Dissertation]. Nanjing, China: Nanjing University of Traditional Chinese Medicine (2015).

[26] Jie Z Qingying Y Jiping H Hongmei L. Clinical observation on the treatment of dysmenorrhea by modified Wenjing decoction combined with acupuncture. Heilongjiang J Trad Chinese Med. (2014) 43:50–1.

[27] Huoman W Zhaoping L Yunqi W Lijuan X. Observation on the efficacy of modified Wenjing decoction combined with warm acupuncture in the treatment of 47 cases of primary dysmenorrhea. Guiding J Trad Chinese Med. (2014) 20:102–4.doi: 10.13862/j.cnki.cn43-1446/r.2014.08.037

[28] Kho KA Shields JK. Diagnosis and Management of Primary Dysmenorrhea. JAMA. (2020) 323:268–9. doi: 10.1001/jama.2019.16921, PMID: 31855238

[29] Algenio-Anciro S Valerio C. Combined Oral contraceptive pills for primary dysmenorrhea. Am Fam Physician. (2024) 109:513–4. PMID: 38905546

[30] Gao SJ Li XL Gao R Tan WH Li W Liu L. Danggui Buxue decoction alleviates primary dysmenorrhea in rats by regulating the MEK1/2/ERK1/2/NF - κB pathway. Fitoterapia. (2024) 180:106315. doi: 10.1016/j.fitote.2024.106315, PMID: 39615702

[31] Shimiao S. Clinical observation on the efficacy of Electroacupuncture at a single Acupoint and Acupoint combination in the treatment of primary dysmenorrhea.[Dissertation]. Changchun, China: Changchun University of Chinese Medicine (2024). doi: 10.13729/j.issn.1671-7813.Z20230362

[32] Lina X Li D. Efficacy of modified Wenjing decoction in the treatment of primary dysmenorrhea of cold stagnation and blood stasis type and its influence on uterine arterial hemodynamics. J Pract Trad Chinese Intern Med. (2024) 38:116–8.

